# Cancer and neurodegeneration: two sides, same coin?

**DOI:** 10.18632/oncotarget.16190

**Published:** 2017-03-14

**Authors:** Sara Garcia-Ratés, Susan Greenfield

**Affiliations:** Neuro-Bio Ltd., Culham Science Center, Oxford, UK

**Keywords:** acetylcholinesterase, alpha7 nicotinic receptor, allosteric modulator, calcium, receptor upregulation

Two papers just published would at first glance have little in common: one features a novel approach to combating metastases [1] whilst the other suggests a new strategy for treating Alzheimer's, [2]. Nonetheless, here we describe a possible link based on the concept that both cancer and neurodegeneration are inappropriately activated forms of development, and in both cases could be mediated by the same bioactive peptide acting at the same receptor.

A fundamental problem in animal biology is how the three major control processes of the body, - the endocrine, immune, and central nervous systems, - achieve the coordination that is essential for the cohesive functioning of the organism. When perturbed, such coordination could be a central, albeit overlooked, factor in the aetiology of many disease states from cancer to neurodegeneration.

Acetylcholinesterase (AChE) has non-classical, non-enzymatic actions that could be pivotal in both brain development and degeneration, this latter process could be an aberrant recapitulation of the former by virtue of the effects of an AChE derived C-terminal peptide, acting at an allosteric site at the alpha-7 nictotinic acetylcholine receptor (α7-nAChR) [3].

Both the α7-nAChR and AChE are present early in embryonic tissues, well before the appearance of the choline acetyltransferase (ChAT) which is required for the synthesis of acetylcholine (ACh). Additionally, recent published papers show the different levels of AChE peptide in different stages of development [4], and in brain of Alzheimer's disease patients [2], where development levels of peptides are recapitulated in AD brains.

Both *in vitro* and *ex vivo* studies show actions of the AChE- peptide along a trophic-toxic spectrum, where neurotrophic effects occur in response to short, low doses of peptide treatment whereas longer exposure result in cell death [3]. Binding of peptide to an allosteric site on the α7-nAChR enhances calcium influx and up-regulation of α7-nAChR expression in neuronal cell cultures [3]. Interestingly enough, a similar signalling system could be operational in metastases: studies with breast cancer cell lines have shown an enhancement of metastatic cell activity in response to peptide treatment, in a calcium and α7-nAChR dependent manner [5]. Most recently we have demonstrated that AChE-pept ide could indeed play a pivotal role in cell migration, with its action blocked by interception at the level of the α7-nAChR [1].

**Figure 1:**
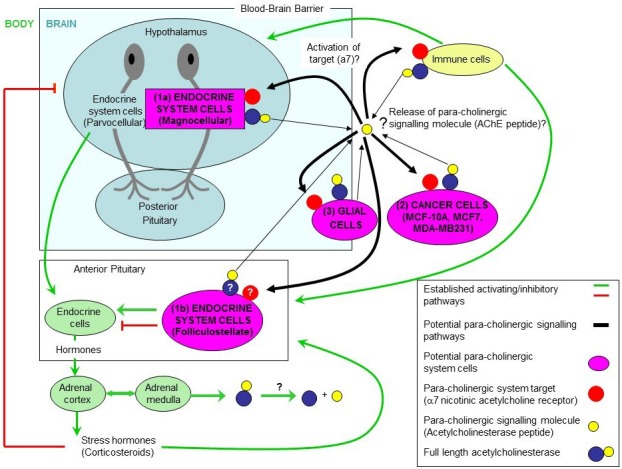
The Para-Cholinergic system shows the relation between the body and the brain areas with the AChE peptide as signalling molecule

AChE is present throughout the brain and body in areas where there is little of its normal substrate ACh, and indeed where there is no neuronal transmission at all. Over the ensuing decades, AChE has been located in a diverse range of non-neuronal cell types, e.g. saliva, placenta, erythrocytes and glia, as well as epithelial, endothelial, immune and cancer cells. Additionally, non-hydrolytic actions of AChE have been widely associated with developmental processes, stress responses, and regulation of apoptotic cell death. This new ‘para-cholinergic’ system (shown in Figure 1) where the AChE peptide would be the signalling molecule, differs from the classic cholinergic in that: is a more generalised mechanism involving body and brain; is depending and limited to the availability of choline (i.e from diet) and the signalling is via an allosteric site on the alpha7 nicotinic receptor linked to an intracellular cascade mediated by calcium; it involves not only the nervous system but also the endocrine and immune system; its action is slow (seconds to minutes instead of milliseconds as in the cholinergic system); the effects are long-term (neurite outgrowth); and the cell type involved in the system not only would include not only neurons but also glia [6], folliculostellate endocrine cells [7], chromaffin cells [8] and cancer cells [5] (summarized in table below).

**Table 1:** Comparison of characteristics of the cholinergic system versus the Para-Cholinergic system

Classic Cholinergic	Para-Cholinergic
Specific functions	More generalised mechanism
Rate limiting factor: ChAT	Rate limiting factors: choline availability, receptor modulation
Primary ligand: acetylcholine	Primary ligand: Choline
Signalling via action potentials	Signalling via intracellular cascades, secreted factors
Fast	Slow
Short-term effects	Long-term effects
CNS, ANS, Neuromuscular junction	Including glia, folliculostellate, chromaffin, cancer cells

In conclusion, a novel signalling system could be operational throughout the brain and body that utilises the familiar components of traditional cholinergic neurotransmission (the alpha-7 receptor and AChE) but is distinguished from it without any further burden on gene expression or protein synthesis, merely requiring as it does the absence of ChAT. The normal substrate ACh would not be needed as the primary ligand for the alpha 7 receptor to be modulated by AChE peptide, could be choline, readily derived from the diet. Hence this form of inter-cellular communication would not be restricted to nervous system signalling and as such could represent a very basic system for triggering calcium entry into a wide range of cells for promoting growth, that would have the further advantage of allowing interfacing between the very distinct control systems (nervous, endocrine and immune). The problem, as illustrated both for metastases [1] and for neurodegeneration [2] would be when it is activated inappropriately.
